# Quantum Plasmonics in Sub-Atom-Thick Optical Slots

**DOI:** 10.1021/acs.nanolett.3c02537

**Published:** 2023-11-29

**Authors:** Jeremy J. Baumberg, Ruben Esteban, Shu Hu, Unai Muniain, Igor V. Silkin, Javier Aizpurua, Vyacheslav M. Silkin

**Affiliations:** † Nanophotonics Centre, Cavendish Laboratory, University of Cambridge, Cambridge CB3 0HE, United Kingdom; ‡ Donostia International Physics Center, P. de Manuel Lardizabal 4, 20018 San Sebastián/Donostia, Basque Country, Spain; § Centro de Física de Materiales, Centro Mixto CSIC-UPV/EHU, P. de Manuel Lardizabal, 5, 20018 San Sebastián/Donostia, Basque Country, Spain; ∥ Tomsk State University, 634050 Tomsk, Russia; ⊥ IKERBASQUE, Basque Foundation for Science, 48009 Bilbao, Basque Country, Spain; ○ Departamento de Polímeros y Materiales Avanzados: Física, Química y Tecnología, Facultad de Ciencias Químicas, Universidad del País Vasco UPV/EHU, 20080 San Sebastián/Donostia, Basque Country, Spain

**Keywords:** plasmonics, quantum, flare, photoluminescence, nanoparticle, nanocavity

## Abstract

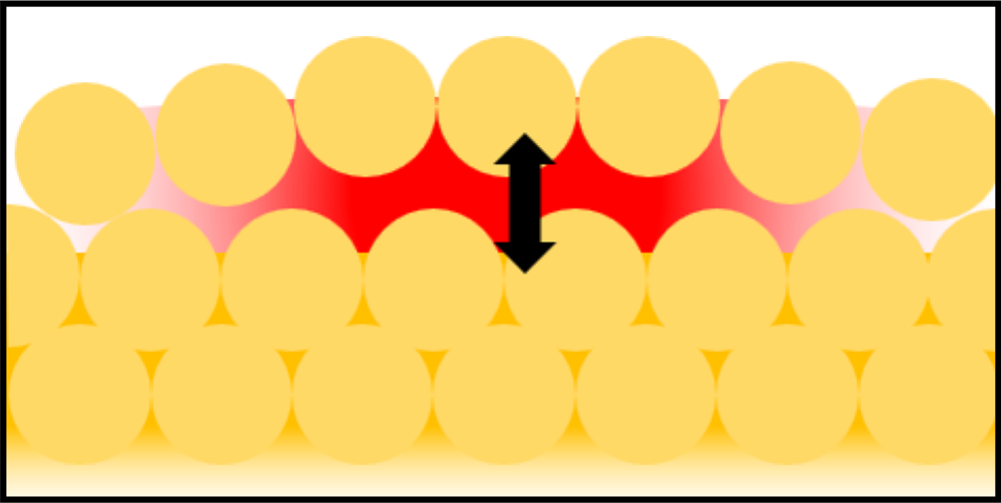

We show using time-dependent density functional theory (TDDFT) that light can be confined into slot waveguide modes residing between individual atomic layers of coinage metals, such as gold. As the top atomic monolayer lifts a few Å off the underlying bulk Au (111), ab initio electronic structure calculations show that for gaps >1.5 Å, visible light squeezes inside the empty slot underneath, giving optical field distributions 2 Å thick, less than the atomic diameter. Paradoxically classical electromagnetic models are also able to reproduce the resulting dispersion for these subatomic slot modes, where light reaches in-plane wavevectors ∼2 nm^–1^ and slows to <10^–2^*c*. We explain the success of these classical dispersion models for gaps ≥1.5 Å due to a quantum-well state forming in the lifted monolayer in the vicinity of the Fermi level. This extreme trapping of light may explain transient “flare” emission from plasmonic cavities where Raman scattering of metal electrons is greatly enhanced when subatomic slot confinement occurs. Such atomic restructuring of Au under illumination is relevant to many fields, from photocatalysis and molecular electronics to plasmonics and quantum optics.

Considerable attention has focused in the past decades on how tightly light can be trapped below its diffraction limit. Exploiting plasmonics and metasurfaces requires metals with free-electron-like properties at the wavelength of interest without interband absorption. Coinage metals such as Au and Ag have been particularly effective at trapping light into nanoscale hot-spots, giving a variety of effects including enhanced Raman scattering (SERS),^1^ second-harmonic generation,^2,3^ photocurrents,^4^ photocatalysis,^5^ and single-emitter strong coupling.^6−8^ Using a plasmonic nanoparticle dimer, the quantum limit for this confinement was ascertained.^9−11^ However, the trapping of light at the metal surfaces of nanoparticles remains subject to numerous questions about the influence of Landau damping, electron spill-out, and tunnelling.^12−15^

Metallic nanostructures can exhibit atomically flat surfaces, with a prototypical example being the (111) face of coinage metals. It is well-known that on such surfaces, in addition to the bulk-like truncated electronic states, so-called surface states emerge.^16^ The surface electronic structure thus consists of two types of electronic states at the Fermi level,^17^ exemplifying a two-component electron system. Pines^18^ first suggested that low-energy plasmons with sound-like (ω_sp_ ∝ *q*, for momentum *q*) long-wavelength dispersion resulted from collective charge motion of such a system with two types of electronic carriers. A similar mode was predicted by Chaplik in the Wigner crystallization of a two-dimensional (2D) electron gas,^19^ whose dispersion (ω_2D_ ∝ √*q*, for in-plane *q*)^20^ transforms into an acoustic one when brought close to a metal surface.^21^ It was later realized that at metal surfaces where a partially occupied quasi-2D surface-state band coexists in the same region of space as an underlying 3D continuum, a well-defined mode with a sound-like dispersion (an acoustic surface plasmon) is formed.^22^ This mode was subsequently observed on Be^23^ and certain noble metals,^23−26^ as well as in graphene deposited onto metal.^27,28^ Bringing graphene close to metals without their touching (e.g., forming gaps spaced by hBN monolayers 0.7 nm thick) produces similar acoustic plasmons.^15^

Here we are concerned with even smaller size gaps, below the diameter of single atoms. We consider how visible frequency acoustic plasmons are produced by a 2D single-monolayer of Au sitting just above 3D bulk Au (Figure 1a,b), which is very different from traditional surface states. Rather than phenomenological theory, we use a full quantum *ab initio* treatment, correctly including all levels of (linear) screening to describe the optical response of this extreme “slot” configuration. Most surprising is the robustness of the obtained trapped plasmons to Ohmic losses and Landau damping. This infiltration of light inside Au surfaces has implications for many fields, including photocatalysis and compact photodetectors.

**Figure 1:**
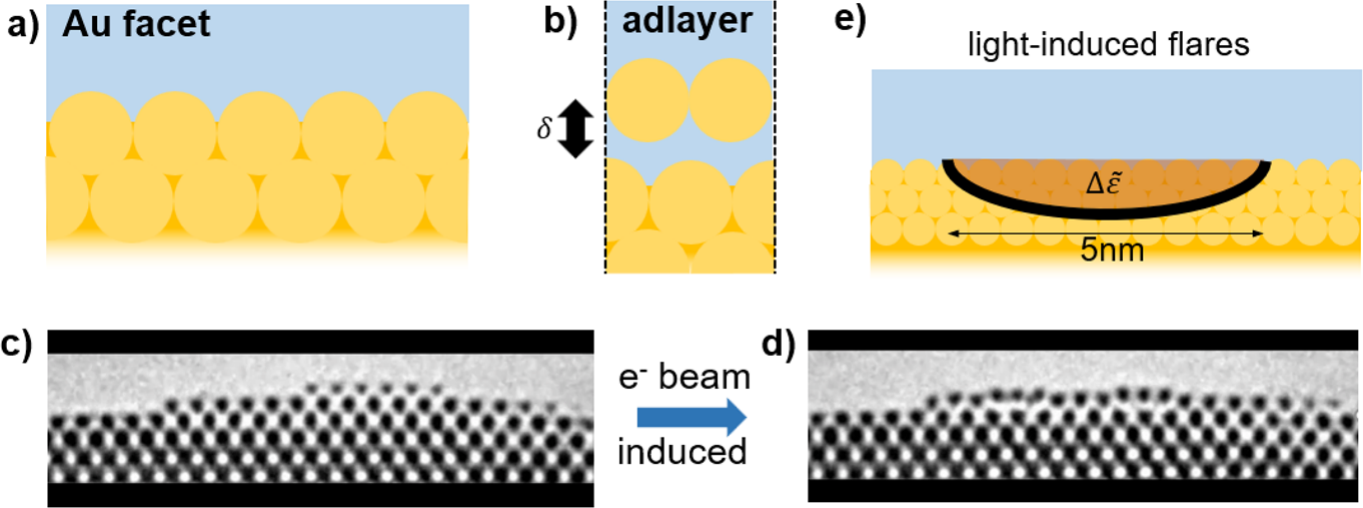
(a) Schematic facet of bulk Au, and (b) with the upper monolayer lifted by δ showing the unit cell. (c, d) TEM frames showing the reconstruction of an edge of a Au nanoparticle under electron beam irradiation. (Reprinted with permission from ref (29). Copyright 2013 Elsevier.) (e) Previous experiment-derived model of complex permittivity change for surface defects forming flares, shown to be ∼5 nm wide.

After developing this theory, we then explore how such slot modes might be observed. Delamination of the top atomic layer of Au (“adlayer”) has already been seen in ultrahigh vacuum (UHV) transmission electron microscopy (TEM) on the edge of Au nanoparticles (Figure 1c,d) induced by high electron beam currents.^29^ As well as individual atoms moving, collective motions of entire surface monolayers are clearly identified, on slow time scales of seconds, which can form atomic-scale slots. On the other hand, spectrally broad flashes of light emitted by visible-pumped plasmonic metal nanostructures, known as “flares”,^30−32^ have been recently identified as arising from surface metal defects ≃5 nm wide (Figure 1e). We examine evidence that these flares may be connected and ultimately originate from subatomic slot modes.

Compared to phenomenological models of nonlocality commonly used, here we perform *ab initio* calculations of the response function of the Au(111) surface, which include the full many-body nonlocal dynamical screening by electrons in the specific atomic-scale configuration of the surface adlayer.^17^ A finite-thickness-layer geometry,^33^ which considers slabs of 21 atomic layers of Au(111), separated by a vacuum equivalent to 8 interlayer spacings, is periodically repeated in the *z* direction to obtain the Kohn–Sham electronic structure using our band structure tool.^34^ In the response calculations, realized with an in-house code,^35^ the adiabatic local density approximation (ALDA) is employed to account for the exchange-correlations. As for surfaces on bulk gold,^36,39^ both ALDA and the random-phase approximation (RPA) give similar results.

The dynamical electronic excitations supported by this system characterized by their dispersion (*q*, ω) can be obtained from the imaginary part of the ALDA surface response function *g*, termed the surface loss function.^37^ The peaks in Im(*g*) determine the energy and lifetime of the surface collective excitations supported in this configuration. When the top monolayer (1 ML) lifts by δ = 2 Å from the semi-infinite Au, thus forming an extreme subatom-thick slot, a new mode (red arrows) is clearly visible in both Re,Im{*g*} at ≃0.35 eV (Figure 2a, with *q* = 0.0093 au here always along ΓM direction), in addition to the standard surface plasmon response near 2.25 eV. At higher *q* = 0.056 a.u., this new mode tunes to 1.66 eV (purple arrow). The nature of this mode becomes clear when the associated charge density is mapped at different energies and positions from the surface (Figure 2b, with *q* = 0.0093 a.u.). As δ increases, a weak spectrally broad mode appears at δ = 1 Å near 1 eV. Above 1.5 Å, a distinct resonance appears at 0.36 eV. For such wider gaps, the 1 ML is already electronically separated from the underlying bulk, with the electron density halfway between the top two atomic planes dropping as exp(−δ/δ_*e*_), δ_*e*_ = 1.5 Å (Figure 2f).

**Figure 2:**
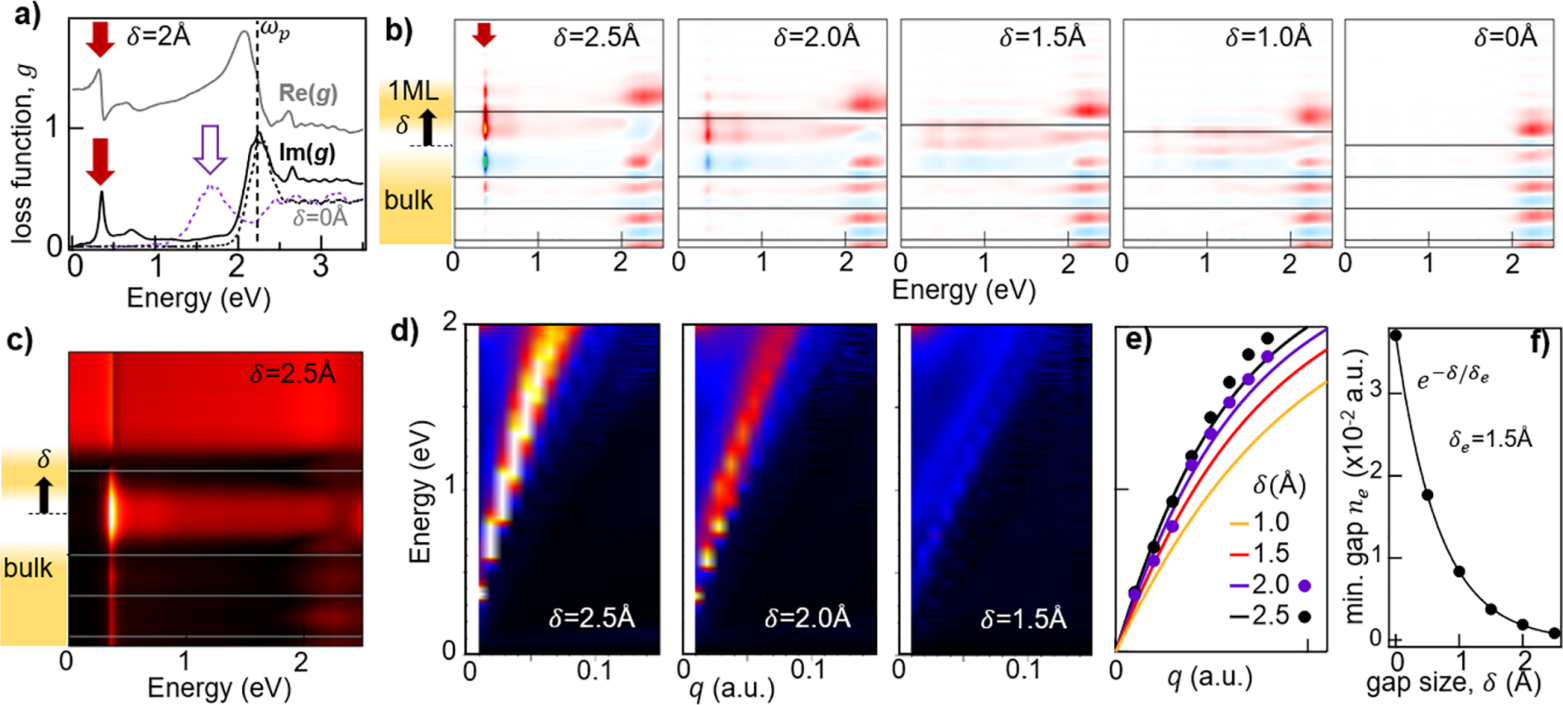
(a) Real and imaginary parts of the surface response function for 1 ML Au separation δ = 2 Å, at in-plane wavevector (along ΓM) *q* = 0.0093 a.u. (solid black and grey), *q* = 0.056 a.u. (dashed purple). Dotted black curve is for semi-infinite Au/air (δ = 0). Arrows mark acoustic plasmon peaks. (b) Induced charge at different excitation energies and depths (horiz. lines show planes of atomic cores) for *q* = 0.0093 a.u. at different 1 ML separations (color scale red-to-blue ±2 a.u.). (c) Field enhancement *E*_*z*_/*E*_0_ obtained from TDDFT calculations for δ = 2.5 Å, *q* = 0.0093 a.u. showing slot mode confinement. (d) Surface loss function Im{*g*} obtained within TDDFT for δ as marked. (e) Dispersions of the acoustic mode (points) extracted from (d) and from classical model predictions (lines) obtained with eq 1. (f) Minimum charge density located halfway between topmost two Au atomic layers vs δ, together with exponential fit.

The induced charge distribution leads to a tightly confined optical field in the slot (Figure 2c) with FWHM 2.0 Å for 1.5 Å gap, smaller than the 3 Å diameter of each Au atom. At δ = 2.5 Å, the slot plasmon survives for up to a few periods (propagating a few plasmonic wavelengths). We calculate the surface excitation spectrum from surface loss function Im[*g*(*q*, ω)] (Figure 2d). The plasmon dispersion relation flattens as the gap decreases (with complex behavior seen below δ = 1.5 Å). The plasmonic excitation occurs for extremely large *q* values, much larger than for a simple metallic substrate or even >1 nm metallic gaps. Thus, TDDFT provides compelling evidence that plasmons can be localized in the crack between layers of Au atoms.

This full ab initio calculation can be compared with analytic solutions for plasmons based on classical local electromagnetic theory. The equivalent structure is a metal–insulator(slot)–metal(monolayer)–insulator or MIMI configuration. For small gaps, the MIMI mode dispersion follows
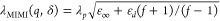
1where a Drude approximation is used for the metal (Au) with background permittivity ε_∞_ = 8.5, insulator permittivity ε_*d*_ = 1, and plasma wavelength λ_*p*_ = 148 nm. Here
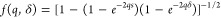
2

The monolayer of Au of thickness *s* = 2.35 Å, set to the (111) layer spacing, is lifted δ = 1–2.5 Å above one of the facets (lines in Figure 2e). This classical dispersion matches the full quantum theory (points in Figure 2e) surprisingly well. The large *q* obtained within both models corresponds to decay lengths into the metal *q*^–1^ ≳ 0.5 nm, matching the *ab initio* optical field distributions (Figure 2c). Previous models also give similar dispersions when including coupling of a 2D surface 1 ML to bulk Au^21^ (see SI for derivation of eq 1 and discussion of other expressions for the dispersion). However, it is remarkable that such local classical models are capable of reproducing much of the full quantum plasmonic effects including dynamical screening and extreme subatomic surface confinement.

The success of classical models in reproducing the *ab initio* acoustic mode properties at δ ≥ 1.5 Å can be rationalized from the calculated electronic structure of all the systems considered here (reported in SI S1). Upon upward displacement of the top Au monolayer by 0.5 Å, only a slight modification in the Au(111) electronic structure can be seen. Once δ increases to 1 Å, a weak broad resonance related to the Au monolayer emerges around the Fermi level. For δ = 1.5 Å this resonance becomes stronger. Nevertheless, a significant portion of the electronic density associated with this resonance resides in the underlying Au(111). We suggest that interband transitions between this resonance and the bulk states do not allow the formation of a well-defined acoustic plasmon mode in this regime of separations (no intermixing of quantum states). In contrast, for δ ≥ 2 Å we find a true quantum-well state^38^ with strong localization in the Au monolayer. Thus, a clear two-component electron system with spatially separated electronic states at the Fermi level is realized. Due to this (and as for a 2D electron gas embedded in a 3D system developed in the literature),^39^ a classical model can be applied in our situation. In summary, classical models work well to describe acoustic modes when there is no intermixing of quantum states of different subsystems in the vicinity of the Fermi level.

We now discuss how such subatomic slot modes might manifest in experiment. Both inelastic electron energy-loss spectroscopy and X-ray photoemission are currently not fast enough to capture the atomic dynamics seen by TEM (Figure 1c,d). On the other hand, optical spectroscopy typically suffers from poor coupling to such tightly confined light because of the several hundred-fold mismatch in *q* between free space and slot modes. One way to overcome these challenges is to exploit the tightly confined light in plasmonic nanocavities, which acts to impedance match between free space and the slot mode (SI). We thus suggest that it is most likely to observe these theoretically robust modes using extreme plasmonic confinement.

Recent experiments in such nanocavities indeed show a variety of unexplained phenomena, including the observation in high-speed dark-field and SERS spectroscopies of transient signals associated with surface defects on metal facets. These defects can extend up to 3–6 nm (Figure 1e).^30−32^ We briefly describe these experiments, suggest how their optical signatures can match those expected from slot modes, and show this is consistent with known binding energies of surface atomic layers.

For maximum optical confinement, we use nm-thick metal–insulator–metal (MIM) gaps formed underneath a nanoparticle-on-mirror (NPoM) construct (Figure 3a).^10^ In this configuration, the Au nanoparticle (NP) acts as an antenna to feed light at resonant wavelengths into the *d* = 1.2 nm gap between the NP lower facet and underlying mirror, where *d* is defined by a self-assembled monolayer of biphenylthiol (BPT) molecules which are rigid, nonreactive, and have no electronic transitions in the visible. This gap can host the detached gold adlayer of nanometric lateral size and thus the 2.35 Å thickslot mode within it. The NPoM nanogap construct traps light at resonance λ_(10)_ seen in dark-field (DF) scattering (Figure 3b, gray). When pumped by a laser at 633 nm varied from 50 to 200 μW, spectrally broad “flares” are sporadically observed (Figure 3b, d, and c signal increases between 5 and 12 s), as well as the persistent Raman peaks from BPT molecules.^30^ Even under identical excitation conditions, flares in different NPoMs vary, and vary in time (Figure 3b−d). Such flares can sometimes last many seconds and show variations in the spectral peak, intensity, and bandwidth (Figure 3c,d). Average flare spectra (over many NPoMs), however, change little with pump power. The spectral widths of the emission events are typically 20–30 nm, while their center wavelengths span a distribution ≃30 nm wide, independent of laser power.

**Figure 3:**
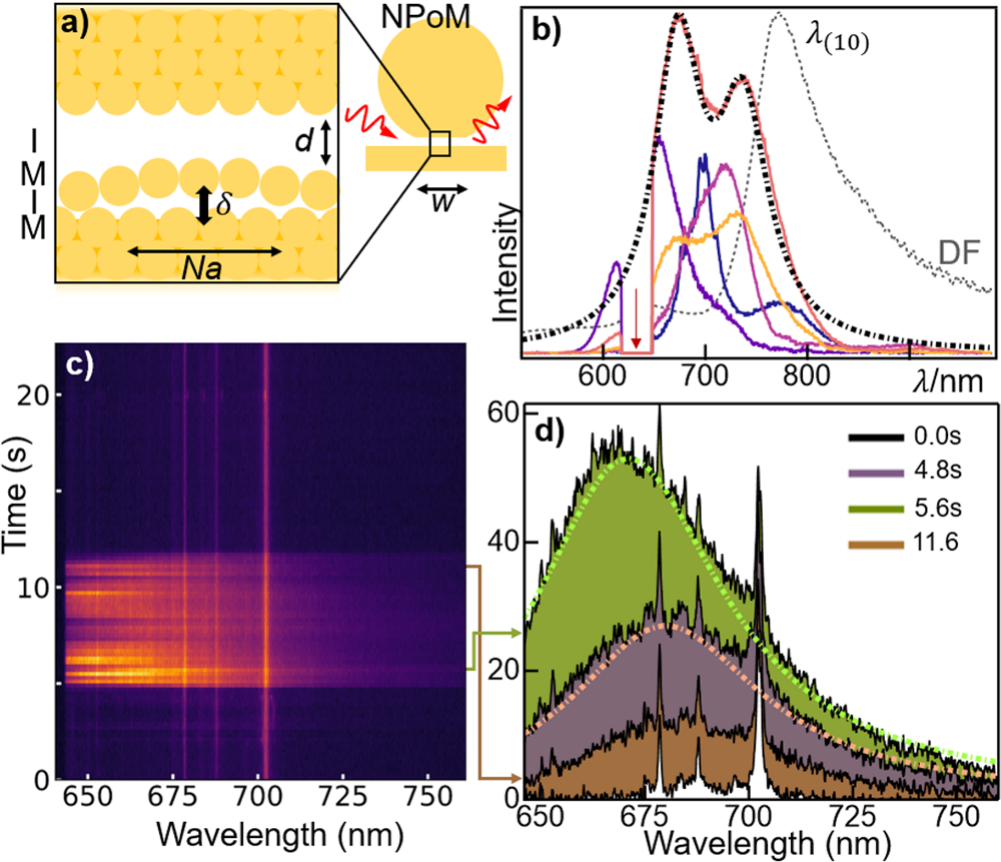
(a) Schematic of 80 nm nanoparticle-on-mirror (NPoM) plasmonic nanocavity with top 1 ML Au lifted distance δ above the lower facet inside the nanogap. Patch width is *Na* for Au atomic diameter *a*. (b–d) Individual flare spectra when CW pumped at 633 nm from either (b) different NPoMs, or (c, d) at different times during the same flare event, 200 ms integration time. Dashed-dot curves are obtained from the model (see text). Vibrational Raman lines in (d) are subtracted from the spectra in (b).

We hypothesize that these flares arise from transient subatomic slot modes created inside the larger NPoM nanogap. Since the slot modes enhance the penetration of light inside the metal (see below), they can enhance light emission (LE) which arises from hot-electron photoluminescence or electronic Raman scattering.^40−42^ In both processes, light excites electrons near the Fermi energy to a higher state, from where they decay back to states on the parabolic *s*-band emitting red-shifted photons that give the flare (Figure 4a). In- and out-coupling of LE from the metal is enhanced by the local optical density of states and thus resonantly enhanced by the slot mode.

**Figure 4:**
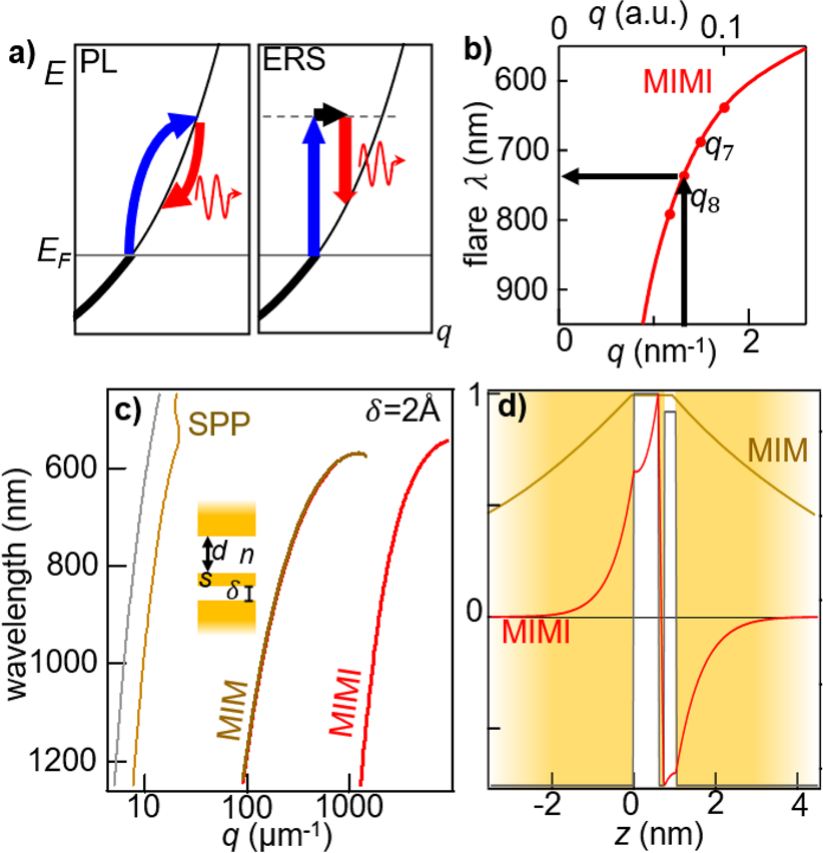
(a) Light emission by hot photoluminescence (PL) and electronic Raman scattering (ERS) processes. In both, electrons near the Fermi energy (*E*_F_) absorb a pump photon, dropping back to the *s*-band by emitting red-shifted photons (flare). (b) Predicted flare peak λ from a *N* = 8 atom-wide patch at δ = 2 Å, *s* = 2.35 Å, *n* = 1. Prediction for *N* = 7 atoms also marked. (c) Classically derived dispersion relation of MIMI, SPP, and MIM modes (see SI) and light line (gray), for *d* = 9 Å, gap *n* = 1.45, *s* = 1.5 Å, δ = 2 Å. The MIMI results in parts b and c are obtained using eq 1. Dispersion of bulk Au surface plasmon polariton-labeled SPP. (d) Corresponding *H*_*y*_ fields (normalized) of MIMI and MIM modes at λ = 650 nm, δ = 3 Å.

We thus derive a simple estimate of the frequency resonance of the slot mode. For Au atomic spacing *a* = 3.0 Å, slot patches of specific lateral size *Na* (Figure 3a) would give lowest-order discrete MIMI modes at wavevector *q*_*N*_ set by lateral quantization^10^ (Figure 4b),
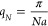
3

Enhanced Lorentzian emission from the NPoM due to the creation of a MIMI slot is expected at wavelength λ_MIMI_ (*q*_*N*_, δ) determined from the mode dispersion (e.g., as given by eq 1). In Figure 3d, we show such emission (dashed lines) given by an average configuration of *N* = 6, with small changes of δ due to fluctuations in time and space of the slot within the gap. The width of this emission spectrum is mainly determined by ε_Au_ (giving *Q* = 17^42^).

The observation of multiple peaks in many flare spectra (Figure 3b) also matches expectations from slot plasmon resonances. Using eqns.(1–3), multiple peaks observed with separation Δλ ≃ 50 nm (Figure 3b, black dashed line) correspond to fluctuations between *N* ≃ 6 ↔ 7 (using δ = 2 Å where the mode becomes strong). This is compatible with TEM observations,^29^ which show just such fluctuations of the atoms at patch ends (Figure 1d). Moreover, the typical time scales found here match those previously seen in TEM dynamics.^29^ The integer variation of atoms within a slot mode patch gives spectral peak switching evident from the discretized MIMI dispersion (Figure 4b). Once a flare has formed (Figure 3d), variations in δ from 2.5 to 2 Å account for Δλ ≃ 30 nm red-shifts of the flare mode, assuming its patch size then remains constant.

The extreme field localization achievable is emphasized in Figure 4c, which compares the classical dispersions of the nanocavity MIM mode and the MIMI subatomic slot-mode. These classical descriptions are validated by the ab initio results (Figure 2d). The wavevector is an order of magnitude larger in the latter, with corresponding 10-fold slowed-down slot mode and strongly enhanced penetration of light into the metal (Figure 4d), which can lead to strong flare emission. We measure the ratio ρ of intensities during flare events to the constant nanocavity LE contribution beforehand, ρ ≃ 20 (Figure 3). Since *ab initio* LE calculations are not yet feasible, evaluation is currently intractable. However, our simulations show strong field enhancements when patches a few atoms wide are separated a few Å from the substrate (SI), further supporting our hypothesis that subatomic slot waveguides may produce these flares.

If subatomic slot modes are responsible for flares, then the rate at which they are created should depend on the cohesive binding energy of the top atomic layer. We thus measure the flare formation rates for a series of >100 NPoMs with different metals as the top monolayer (Figure 5). These atomic monolayers are created by well-known underpotential electrochemical deposition using the flat Au as the working electrode, and verified elsewhere.^43^ Indeed we find that the flare formation rates depend significantly on the top layer of metal. While 1 ML Ag produces similar flares but at higher rates, Pd blocks flare production, as suggested by the adatom cohesive energies on these surfaces.^44,45^ We also find that flare production rates (but not their spectral characteristics, see Figure S7) depend on the molecular species in the nanogap, and that fewer are generated on (100) than (111) Au surfaces, again matching known energy barriers.^46^ As a result, for nanodecahedra on mirror (NDoM) constructs (Figure 5c), which only possess (111) facets,^47^ the rates are on average faster than for NPoMs with a mixture of (100) and (111) facets. We emphasize that similar results are observed for picocavities.^48^

**Figure 5:**
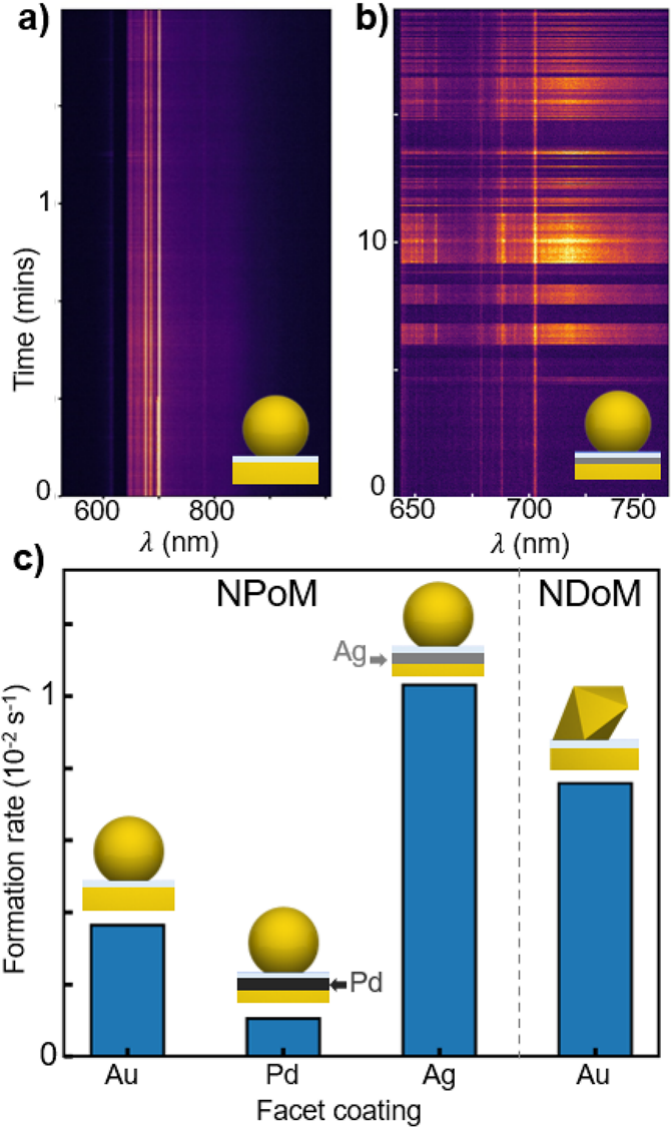
(a) NPoM light emission vs time from all-Au facets under 5 μW·μm^–2^ laser intensity at 633 nm. (b) NPoM light emission vs time from 1 ML Ag coated Au facet under 5 μW·μm^–2^ laser intensity at 633 nm. (c) Relative flare formation rates under 130 μW·μm^−2^ for NPoMs with Au, Pd, and Ag top monolayers, as well as a nanodecahedra-on-mirror (NDoM) Au facet possessing only (111) facets (see SI).

We further stress the connection between subatomic thick slots and single adatoms known as “picocavities”, which also are found to locally enhance optical fields.^10^ In our picture the fast appearance, disappearance, and modulation of flares corresponds to the opening of, and fluctuations in, subatomic slot gaps, and in the number of atoms in each patch of suspended monolayer. Tracking these flares can thus open up a way to access surface modes on metals in ambient conditions, similar to that of inelastic spectroscopy of correlated electronic states in 2D electron gases^49−51^ as well as to characterize atomic-scale structural modifications in metallic nanogaps. Particularly interesting is studying the effects of lattice symmetry, metal type, and gap contents (Figures S7 and S8). Ab-initio models are thus now available to predict how these would influence the light confinement and coupling to the metal. Specifically, it is now important to theoretically investigate the optical forces associated with such subatomic slot modes, and their stability.

In summary, we show theoretically that light can be trapped in subatomic wide slots between a monolayer and bulk gold. The acoustic dispersion revealed is modulated by the slot gap and creates the most tightly confined visible light yet (previously one or several atoms spanned the gap^15,52^). We show that the slot mode can be surprisingly well described in a classical approximation at separations exceeding 1.5 Å when a true quantum-well state is formed in the Au monolayer at the Fermi level. The power of our ab initio calculations is that they include full screening and surface damping effects. We suggest how this slot mode might be responsible for the flares observed in inelastic light scattering experiments, feeding intense optical fields into the metal to drive the electronic light emission. This provides intriguing directions for future experiments and theory on the interaction of light and molecule–metal surfaces.

## Supporting Information Available

S1: Subatomic slot waveguide modes. S1.1: Calculation of the classical loss function. S1.2: Loss function calculated in the ab initio TDDFT framework. S1.3: Comparison of classical and TDDFT calculations. S2: Classical dispersion of the MIMI mode. S2.1: Approximated dispersion. S2.2: Dispersion when treating the metal layer as a 2D material. S2.3: Dispersion of MIM structure and surface plasmon. S3: Classical electromagnetic simulations of a NPoM with an atomic-thick patch. S4: Experimental measurement of flares (PDF)
