# Supplementary material for: Quantum Plasmonics in Sub-Atom-Thick Optical Slots
Source: Nano Lett. 2023 Nov 29;23(23):10696–702. doi: 10.1021/acs.nanolett.3c02537 (PMC10722603; doi:10.1021/acs.nanolett.3c02537)
Supplement: Supplementary file 1 — nl3c02537_si_001.pdf [file nl3c02537_si_001.pdf]

Supplementary Information
Quantum plasmonics in sub-atom-thick optical slots

Jeremy J Baumberg[1], Ruben Esteban[2,3], Shu Hu[1], Unai Muniain[3], Igor V Silkin[4], Javier Aizpurua[2,3], and Vyacheslav M Silkin[3,5,6]

[1] *Nanophotonics Centre, Cavendish Laboratory, University of Cambridge, Cambridge CB3 0HE, UK*
[2] *Centro de Física de Materiales, Centro Mixto CSIC-UPV/EHU, P. de Manuel Lardizabal, 5, 20018 San Sebastián/Donostia, Basque Country, Spain*
[3] *Donostia International Physics Center, P. de Manuel Lardizabal 4, 20018 San Sebastián/Donostia, Basque Country, Spain*
[4] *Tomsk State University, 634050 Tomsk, Russia*
[5] *Departamento de Polímeros y Materiales Avanzados: Física, Química y Tecnología, Facultad de Ciencias Químicas, Universidad del País Vasco UPV/EHU, 20080 San Sebastián/Donostia, Basque Country, Spain*
[6] *IKERBASQUE, Basque Foundation for Science, 48009 Bilbao, Basque Country, Spain*

S1 Sub-atomic slot waveguide modes

S1.1 Calculation of the classical loss function

We first derive the classical non-retarded loss function of the Metal-Insulator-Metal-Insulator (MIMI) planar structure as depicted in Figure S1a. A thin gold layer of thickness $s = 2.35$ Å is situated at a distance δ above a semi-infinite gold substrate (forming the two metal layers 'M' in the MIMI structure). The material on both sides of the thin gold layer is vacuum (insulator 'I', both dielectric layers) with (relative) permittivity $\varepsilon_d = 1$. The gold is described by the experimental classical (relative) permittivity $\varepsilon_{\mathrm{Au}}$ [1], except where noted otherwise. We consider a nonretarded description because the parallel wavevector q of the MIMI plasmonic mode is very

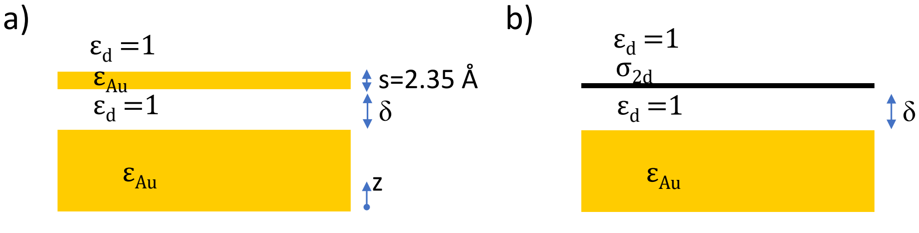

Figure S1: Sketch of the systems considered, used to obtain dispersion relations. (a) MIMI configuration, with a thin gold layer of thickness $s = 2.35$ Å separated from a gold semi-infinite substrate below by a vacuum gap of thickness δ. The material above the thin metal is also vacuum. (b) Same system after substituting the thin metal layer by a 2D material of same conductivity. The z-direction perpendicular to the interfaces is shown in (a), with $z = 0$ at the top of the monolayer.

large, $q \gg k_{\mathrm{Au}}$ and $q \gg k_{\mathrm{d}}$ (k_{Au} and k_{d} are the magnitudes of the wavevector in gold and in vacuum, respectively). The direction normal to the layers is defined as the z axis, with $z = 0$ the top of the monolayer, and $z = -s - \delta$ the top of the substrate.

The loss function corresponds to $\mathrm{Im}[g(\mathbf{q}, \omega)]$, the imaginary part of the surface response function g. Here $g(\mathbf{q}, \omega)$ depends on the in-plane momentum $\mathbf{q}$ and energy ω, and is defined [2] by equation[i]

$$g(\mathbf{q}, \omega) = \int n^{\mathrm{ind}}(\mathbf{r}, \mathbf{q}, \omega) e^{qz}\, d\mathbf{r}, \tag{S1}$$

where $n^{\mathrm{ind}}(\mathbf{r}, \mathbf{q}, \omega)$ is the electronic density induced by an external potential of the form

$$V^{\mathrm{ext}}(\mathbf{r}, \mathbf{q}, \omega) = -\frac{2\pi}{q} e^{qz} e^{i\mathbf{q}\cdot\mathbf{r}_{\parallel}} e^{-i\omega t}, \tag{S2}$$

which corresponds to the 2D Fourier transform of the Coulomb potential created by a point charge placed above the system at an infinite distance, with the charge oscillating in time as $e^{-i\omega t}$. We obtain next the potential and charges induced by this external potential $V^{\mathrm{ext}}(\mathbf{r}, \mathbf{q}, \omega)$. In each of the four layers of the system, the permittivity is homogeneous, and thus we need to impose the boundary conditions only at the three vacuum-gold interfaces. We start by writing the total potential of the layer j as

$$V_j(\mathbf{r}, \mathbf{q}, \omega) = -\frac{2\pi}{q} \{v_{j+}(\mathbf{q}, \omega) e^{qz} + v_{j-}(\mathbf{q}, \omega) e^{-qz}\} e^{i\mathbf{q}\cdot\mathbf{r}} e^{-i\omega t}. \tag{S3}$$

For $j = 1$ (top vacuum layer) $v_{1+} = 1$, because this term corresponds to the external potential of Eq. (S2). Further, for $j = 4$ (gold substrate) $v_{4-} = 0$, so that the fields are finite in the limit $z \to -\infty$. The rest of the coefficients are obtained by imposing the continuity of the potential $V(\mathbf{r}, \mathbf{q}, \omega)$ and of the normal component of the displacement field $D_z(\mathbf{r}, \mathbf{q}, \omega) = -\varepsilon(\mathbf{r}, \omega) \frac{\partial V(\mathbf{r}, \mathbf{q}, \omega)}{\partial z}$ across the interfaces at $z = 0$, $z = -s$ and $z = -(s+\delta)$. The following set of equations is obtained

$$1 + v_{1-} = v_{2+} + v_{2-}, \tag{S4a}$$

$$v_{2+} e^{-qs} + v_{2-} e^{qs} = v_{3+} e^{-qs} + v_{3-} e^{qs}, \tag{S4b}$$

$$v_{3+} e^{-q(s+\delta)} + v_{3-} e^{q(s+\delta)} = v_{4+} e^{-q(s+\delta)}, \tag{S4c}$$

$$1 - v_{1-} = \varepsilon_{\mathrm{Au}}(\omega)(v_{2+} - v_{2-}), \tag{S4d}$$

$$\varepsilon_{\mathrm{Au}}(\omega)(v_{2+} e^{-qs} - v_{2-} e^{qs}) = v_{3+} e^{-qs} - v_{3-} e^{qs}, \tag{S4e}$$

$$v_{3+} e^{-q(s+\delta)} - v_{3-} e^{q(s+\delta)} = \varepsilon_{\mathrm{Au}}(\omega) v_{4+} e^{-q(s+\delta)}. \tag{S4f}$$

With the solutions of this system of equations, we can calculate the charge density induced by the potential from the polarization vector $\mathbf{P}(\mathbf{r}, \mathbf{q}, \omega) = -\{\varepsilon(\mathbf{r}, \omega) - 1\} \nabla V(\mathbf{r}, \mathbf{q}, \omega)$. In contrast to the TDDFT calculations, in the classical system there is no induced volumetric charge density, because $n^{\mathrm{ind}}_{\mathrm{vol}}(\mathbf{r}, \mathbf{q}, \omega) = -\nabla \cdot \mathbf{P}(\mathbf{r}, \mathbf{q}, \omega) = 0$, as can be obtained from Eq. S3. Therefore, there is only the surface charge density induced at the interfaces, calculated as $n^{\mathrm{ind}}(\mathbf{r}, \mathbf{q}, \omega) = \mathbf{P}(\mathbf{r}, \mathbf{q}, \omega) \cdot \hat{\mathbf{n}}_{\mathrm{out}}$, where $\hat{\mathbf{n}}_{\mathrm{out}}$ is a unitary vector pointing outwards from the metallic regions. We calculate the induced density to be

$$\begin{aligned} n^{\mathrm{ind}}(\mathbf{r}, \mathbf{q}, \omega) &= P_z(0, \mathbf{q}, \omega)\delta_{\mathrm{D}}(z) - P_z(-s, \mathbf{q}, \omega)\delta_{\mathrm{D}}(z+s) + P_z(-s-\delta, \mathbf{q}, \omega)\delta_{\mathrm{D}}(z+s+\delta) \\ &= \frac{\varepsilon_{\mathrm{Au}}(\omega) - 1}{2} \Big[(v_{2+} - v_{2-})\delta_{\mathrm{D}}(z) - (v_{2+} e^{-qs} - v_{2-} e^{qs})\delta_{\mathrm{D}}(z+s) \\ &\qquad + v_{4+} e^{-q(s+\delta)} \delta_{\mathrm{D}}(z+s+\delta) \Big], \end{aligned} \tag{S5}$$

[i]Equations in Sec. S1 are in atomics units if not stated otherwise.

where the symbol δ_{D} is the Dirac delta function. By inserting this expression into Eq. (S1), we obtain

$$g(\mathbf{q},\omega)=\frac{\varepsilon_{\mathrm{Au}}(\omega)-1}{2}[v_{2+}(1-e^{-2qs})+v_{4+}e^{-2q(s+\delta)})]. \tag{S6}$$

Last, by replacing the solutions of Eq. (S4) in this expression, the classical surface response function of the system is found to be

$$g(\mathbf{q}_{\|},\omega)=\frac{\varepsilon_{\mathrm{Au}}(\omega)-1}{\varepsilon_{\mathrm{Au}}(\omega)+1}\frac{(e^{2qs}-1)[(\varepsilon_{\mathrm{Au}}(\omega)-1)^2-e^{2q\delta}(\varepsilon_{\mathrm{Au}}(\omega)+1)^2]-4\varepsilon_{\mathrm{Au}}(\omega)}{(e^{2qs}+e^{2q\delta}-1)(\varepsilon_{\mathrm{Au}}(\omega)-1)^2-e^{2q(s+\delta)}(\varepsilon_{\mathrm{Au}}(\omega)+1)^2}. \tag{S7}$$

S1.2 Loss function calculated in the *ab initio* Time-Dependent Density Functional Theory framework

To evaluate the surface response function from first principles we employed a supercell geometry. The electronic structure of a truncated crystal is simulated considering slabs of finite thickness. A bare Au(111) surface is described by a slab consisting of 21 atomic layers taking the lattice parameter a=4.0647 Å. Since the relaxation of the top atomic layers has marginal effect on the Au(111) surface excitation spectra [3], the calculations were realized with the ideal atomic positions. To form the MIMI structure, the outer atomic monolayer is displaced by a distance δ (the gap separation) relative to its bulk position. To keep the symmetry, both the top and bottom monolayer of the 21-layer slab are displaced. The vacuum separation between slabs (i.e. between the top monolayer of one slab and the bottom one of the next one) corresponds to 7 interlayer spacings. The self-consistent band structure calculations in a local-density approximation (LDA) [4] scheme were realized using an in-house code [5] that uses the norm-conserving electron-ion pseudopotential [6]. The exchange-correlation potential was considered taking the LDA Ceperley-Alder representation [4]. The relativistic effects for the valence states were treated in the scalar relativistic approximation. In the case of bulk gold it was demonstrated [7] that the spin-orbit coupling has limited effect on the dielectric function in the energy region of interest here. As the energy position of the valence d electronic states in Au is not reproduced correctly in the LDA approach, we modified the evaluated one-particle energies following the numerical procedure found in Ref. [8]. This procedure consists of downward shifting the LDA valence d states from the calculated positions to the measured location of their absorption edge and at the same time leaving the s-p states below and above the Fermi level unchanged.

Figure S2 shows the electronic structure calculated in the vicinity of the Fermi level for (a) a bare Au(111) surface represented by a 21-monolayer film and (b-f) the MIMI structure with (b) δ=0.5 Å, (c) δ=1.0 Å, (d) δ=1.5 Å, (e) δ=2.0 Å, and (f) δ=2.5 Å. The red lines in the figure represent the Shockley surface state with strong localization slightly over the Au(111) top atomic layer of the bare gold surface. The Shockley surface state is largely localized over the detached monolayer at short separation distances δ, but its wave function gradually redistributes from this monolayer to the underneath Au(111) surface as δ increases. At separation distance δ=2.5 Å the spatial localization of this state almost entirely coincides with the one obtained for a bare Au(111) surface, with negligible presence in the detached Au monolayer. Regarding new possible excitations in the MIMI structure, it is hard to detect any signature of a resonance related to the top Au monolayer for the smallest δ considered (Figure S2b; δ=0.5 Å). After increasing the gap separation to δ=1.0 Å (Figure S2c), a weak broad resonance not present in the bare Au surface can be identified, marked in the dispersion by pale yellow regions. This resonance becomes well defined already for δ=1.5 Å (marked in yellow in Figure S2d). Last, in Figure S2e,f, corresponding to gap separation δ=2.0 Å and δ=2.5 Å, we find a well-defined quantum-well state with strong localization in the shifted Au monolayer, highlighted by orange lines.

We obtain the density response function of the interacting electron system $\chi(\mathbf{r},\mathbf{r}',\omega)$ in the framework of time-dependent density functional theory [9, 10]. In reciprocal space, χ is a

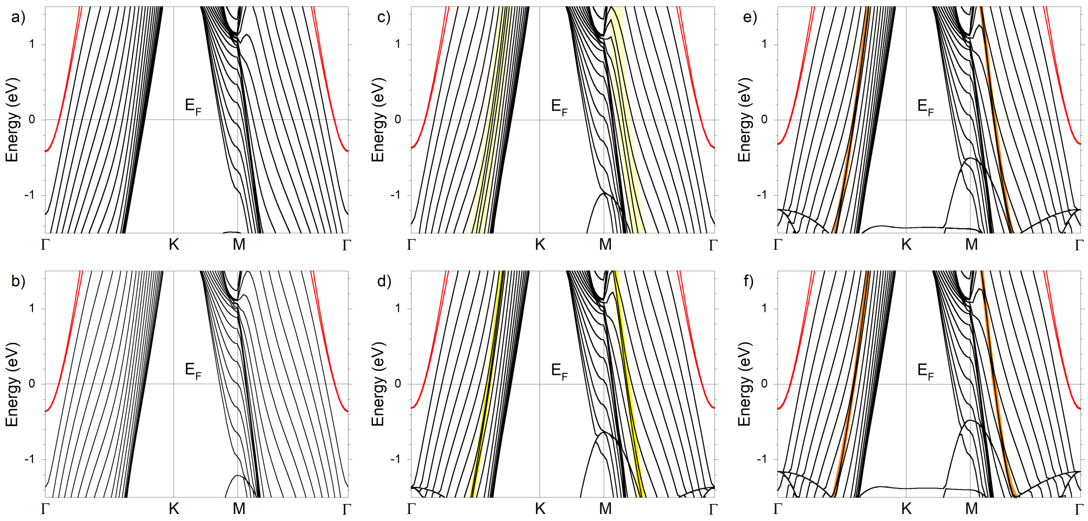

Figure S2: Electronic structure of a bare gold surface and MIMI structures, shown near the Fermi level.The electronic structure is obtained for (a) a bare, 21-atom thick Au(111) slab and (b-f) the MIMI structure described in the text with gap separation (b) δ=0.5 Å, (c) δ=1.0 Å, (d) δ=1.5 Å, (e) δ=2.0 Å, and (f) δ=2.5 Å. The red lines indicate the Shockley surface state, and the pale yellow regions and orange lines correspond to resonances not present in the bare gold surface.

function of the in-plane momentum $\mathbf{q}$ and energy ω and it transforms into the matrix that is obtained from the matrix equation

$$\chi_{\mathbf{GG}'}(\mathbf{q},\omega) = \chi^o_{\mathbf{GG}'}(\mathbf{q},\omega) + \sum_{\mathbf{G}_1,\mathbf{G}_2} \chi^o_{\mathbf{GG}_1}(\mathbf{q},\omega)[V_{\mathbf{G}_1}(\mathbf{q})\delta_{\mathbf{G}_1\mathbf{G}_2} + K^{xc}_{\mathbf{G}_1\mathbf{G}_2}(\mathbf{q},\omega)]\chi_{\mathbf{G}_2\mathbf{G}'}(\mathbf{q},\omega). \quad \text{(S8)}$$

Here $V_{\mathbf{G}_1}$ is the Fourier transform of the bare Coulomb potential. The kernel $K^{xc}_{\mathbf{G}_1\mathbf{G}_2}$ accounts for the exchange-correlation effects. In the present work, we employed two forms for $K^{xc}_{\mathbf{G}_1\mathbf{G}_2}$, namely the adiabatic local-density approximation (ALDA) [10] and the random-phase approximation (RPA) where it is set to zero. Like in bulk gold [11], the calculated excitation spectra obtained with both approximations are very similar. For concreteness, we report data derived with ALDA. χ^o is the response function of the noninteracting Kohn-Sham electrons. To evaluate it, the surface Brillouin zone with full inclusion of the matrix elements was sampled on a 144×144 grid using our own code [12]. All the valence occupied and unoccupied energy bands with energies up to 25 eV above the Fermi level were included. In the present calculations we expanded the χ^o and χ matrices over 151 $\mathbf{G}$ vectors, which point in the perpendicular z direction only since the in-plane local-field effects have limited impact on the surface excitation spectra [13].

After the density response function χ is calculated, the induced charge density is directly obtained by performing the integral

$$n^{\text{ind}}(\mathbf{r},\mathbf{q},\omega) = \int \chi(\mathbf{r},\mathbf{r}',\mathbf{q},\omega)V^{\text{ext}}(\mathbf{r}',\mathbf{q},\omega)d\mathbf{r}'. \quad \text{(S9)}$$

V^{ext} is defined in Eq. (S2). This induced density allows one to obtain the electric fields in Figure 2 of the main text and in Figure S4 below, by solving the Poisson equation $\nabla^2 V(\mathbf{r},\mathbf{q},\omega) = n^{\text{ind}}(\mathbf{r},\mathbf{q},\omega)$.

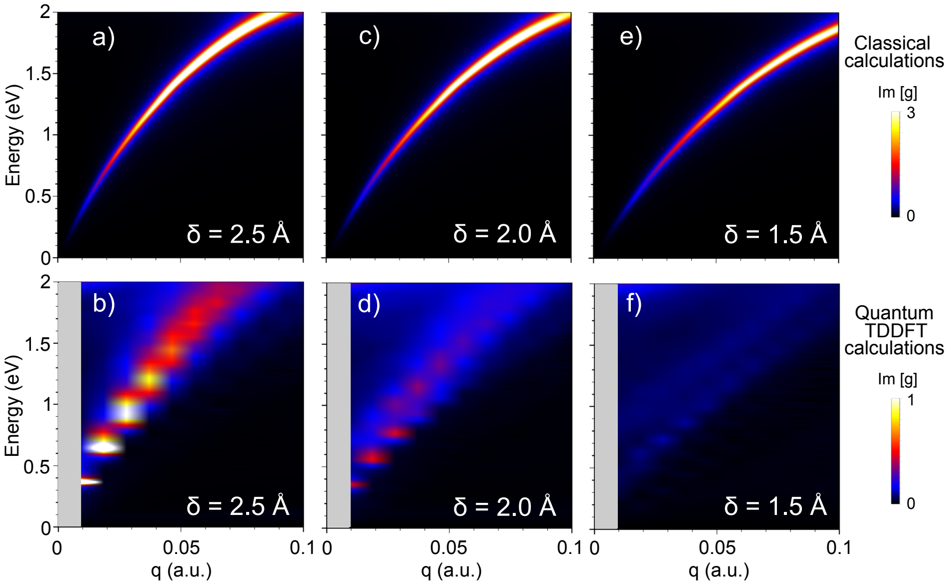

Figure S3: Comparison of the classical and quantum results for the loss function. (a) Classical loss function, given by Eq. (S7), for a gold monolayer separated by $\delta = 2.5$ Å. (b) Loss function obtained from the TDDFT calculations, for $\delta = 2.5$ Å. The gray area indicates the momenta q that are not accessible to the TDDFT calculations. (c,d) Equivalent to (a,b) but for $\delta = 2$ Å. (e,f) Equivalent to (a,b) but for $\delta = 1.5$ Å.

S1.3 Comparison of classical and TDDFT calculations

We compare in Figure S3 the loss function $\mathrm{Im}[g(\mathbf{q},\omega)]$ obtained from the classical surface response function (Eq. (S7)) with that obtained from atomistic calculations based on Time-Dependent Density Functional Theory (TDDFT). The loss function allows us to extract the modes of the system by selecting the energies ω where the loss function is maximum for a fixed momentum q. In particular, the maxima of $\mathrm{Im}[g(\mathbf{q},\omega)]$ obtained from Figure S3 follows the dispersion of the MIMI mode. For the largest gap that we analyze in this work, $\delta = 2.5$ Å, we observe that the classical simulations (panel a) follow a similar dispersion $\omega(q)$ to the TDDFT results (panel b). Further, by reducing the gap size to $\delta = 2.0$ Å (panels c and d) and $\delta = 1.5$ Å (panels e and f), the classical and the atomistic descriptions lead to a similar shift of the MIMI mode towards smaller energy (redshift) for all q.

The difference between both models is larger when considering the absolute values of the loss function. In the classical calculations, the value of the loss function $\mathrm{Im}[g(\mathbf{q},\omega)]$ at the resonant energy corresponding to the MIMI mode increases together with momentum q, and the value of the loss function is similar for all gap sizes. In contrast, the atomistic TDDFT loss function has a clear peak associated to the MIMI mode for small q, while for larger momenta the peak becomes less intense and broader, indicating that the damping of the mode increases considerably. Further, the TDDFT description of the system predicts that the properties of the mode have a stronger dependence on δ as compared to the classical calculations. In particular, for $\delta = 1.5$ Å we observe that the peak of the TDDFT loss function is highly suppressed even for small q (Figure S3), which shows that the MIMI mode becomes heavily damped if the gap is not thick enough. Since the damping of the MIMI mode remains little changed for different δ according to classical calculations, TDDFT calculations become necessary for small gaps.

We can also use a classical description to calculate the electric field distribution of the MIMI mode. We plot in the top row of Figure S4 the classical electric field, $E_z(z,\mathbf{q},\omega) = -\frac{\partial V(z,\mathbf{q},\omega)}{\partial z}$,

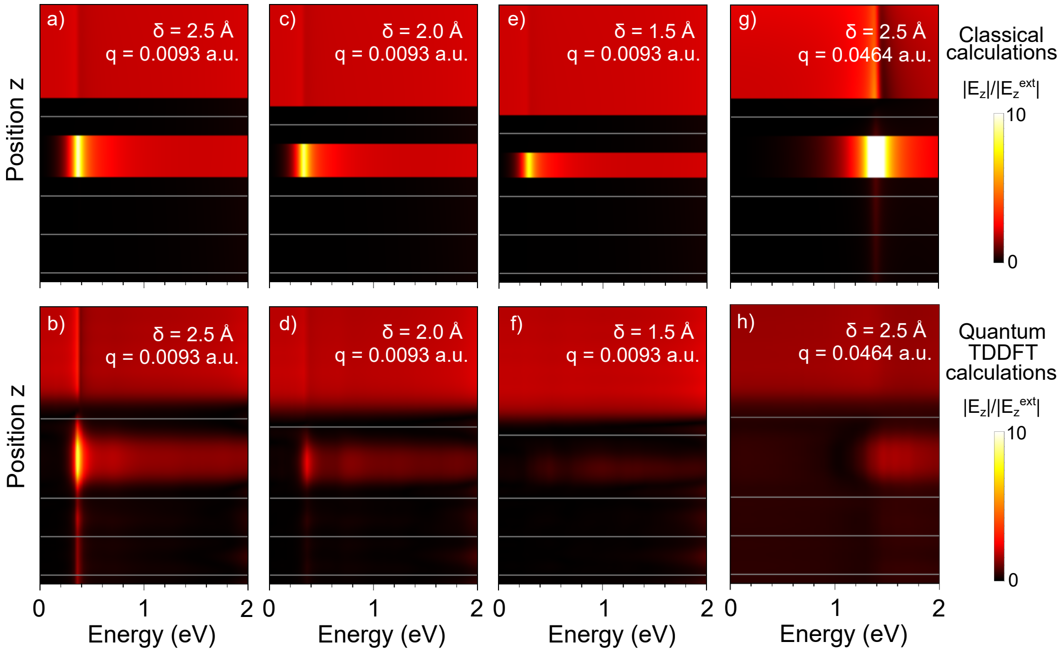

Figure S4: Comparison of the electric field distribution obtained using classical and TDDFT calculations. (a) Distribution along the z axis of the enhancement of the z component of the electric field $|E_z|$, obtained with classical calculations as a function of energy, for $q = 0.0093$ a.u. and $\delta = 2.5$ Å. (b) Distribution along the z axis of the enhancement of the z component of the electric field $|E_z|$, obtained from the TDDFT atomistic calculations as a function of energy, for $q = 0.0093$ a.u. and $\delta = 2.5$ Å. The fields in (a,b) are normalized by the z component of the incident field $|E_z^{\mathrm{ext}}|$ for each energy. (c,d) Same as in (a,b) but for $\delta = 2$ Å. (e,f) Same as in (a,b) but for $\delta = 1.5$ Å. (g,h) Same as in (a,b) but for $q = 0.0464$ a.u. The gray lines in all panels represent the centre positions of each atomic layer in the TDDFT simulations.

where $V(\mathbf{r},\mathbf{q},\omega)$ is the potential obtained from Eqs. (S3) and (S4). The fields are plotted after normalization with respect to the external field $E_z^{\text{ext}}(z,\mathbf{q},\omega) = -\frac{\partial V^{\text{ext}}(z,\mathbf{q},\omega)}{\partial z}$, with the external potential $V^{\text{ext}}(z,\mathbf{q},\omega)$ given by Eq. (S2). For the largest gap of $\delta = 2.5$ Å, Figure S4a shows that for momentum $q = 0.0093$ a.u. (the smallest value that we consider in the TDDFT simulations) there is a classical field enhancement of around $|E_z|/|E_z^{\text{ext}}| \simeq 10$ at energies close to the MIMI mode. For comparison, we show in Figure S4b the corresponding electric field obtained from the TDDFT calculations, after solving the Poisson equation $\nabla^2 V(\mathbf{r}) = n^{\text{ind}}(\mathbf{r})$. The agreement with classical calculations is generally good, with a similar field enhancement for the gap mode that appears at the same energy, although the TDDFT calculations give a narrower peak of the field enhancement plotted as a function of energy. Another important difference lies in the penetration of the field in the first atomic layers of the bulk substrate, which is not captured by classical calculations as they assume an abrupt change of the permittivity at the gap-substrate interface.

The difference between the TDDFT and classical calculations grows as the gap becomes narrower. The classical field enhancements at the MIMI mode energy do not vary much as δ is decreased (from $|E_z|/|E_z^{\text{ext}}| \simeq 10$ with $\delta = 2.5$ Å in Figure S4a to $|E_z|/|E_z^{\text{ext}}| \simeq 8$ with $\delta = 1.5$ Å in Figure S4e), but the TDDFT results show a strong dependence of the electric field on δ. Already for a gap $\delta = 2.0$ Å, despite good agreement between both models regarding the MIMI mode energy (≈ 0.35 eV), the TDDFT calculations (Figure S4d) predict a considerably weaker field enhancement in the gap than the classical results (Figure S4c). This disagreement is more substantial for $\delta = 1.5$ Å (panels e and f), where for the atomistic description of the system the gap mode is not appreciable in the field distribution. Last, we plot in Figures S4g and S4h the field enhancement calculated using the classical and TDDFT descriptions, respectively, for $q = 0.0464$ a.u. and gap width $\delta = 2.5$ Å, to show that the disagreement between the two calculations is considerable for large momenta even for the largest gap of $\delta = 2.5$ Å. The energy of the gap mode is again well recovered by the classical calculations, but the field enhancement is larger. This could imply that for large momenta a non-local permittivity $\varepsilon(\mathbf{q},\omega)$ should be used in the classical approach.

S2 Classical dispersion of the MIMI mode

The exact classical non-retarded dispersion of the Metal-Insulator-Metal-Insulator MIMI waveguide (Sec. S1.1 and Figure S1a) can be obtained from the poles of Eq. (S7). The pole $\varepsilon_{Au} = -1$ corresponds to the (non-retarded) surface plasmon of the semi-infinite gold substrate. The dispersion of the MIMI mode is then given by the equation[ii]

$$1 - \left(\frac{\varepsilon_{\text{Au}} - \varepsilon_{\text{d}}}{\varepsilon_{\text{Au}} + \varepsilon_{\text{d}}}\right)^2 \left[1 - \left(1 - e^{-2q\delta}\right)\left(1 - e^{-2qs}\right)\right] = 0, \tag{S10}$$

with $\varepsilon_d = 1$, which can be solved numerically. The same dispersion can be obtained using the full retarded expression by approximating $k_{\text{d,z}} \approx k_{\text{Au,z}} \approx iq$, where $k_{\text{d,z}}$ and $k_{\text{Au,z}}$ are the components of the wavevector perpendicular to the surfaces in the insulator (i.e. vacuum) and in gold, respectively. This approximation is justified by the large values of the parallel wavevector q (additional discussion in Sec. S2.1.4).

The results are given by the black line in Figure S5a-c, and show an approximately linear dependence of the energy of the mode with q for low energies, and a decreasing slope for larger energies (not appreciable in the figure, the dispersion becomes non-linear for very small energies $\lesssim 0.05$ eV, which we attribute to the imaginary part of ε_{Au}). In all the results plotted in this section, we fix $\delta = 2$ Å. The black line in Figure S5 is thus the same as the violet line in Figure 2e of the main text.

[ii] Equations in Sec. S2 are in SI units if not stated otherwise.

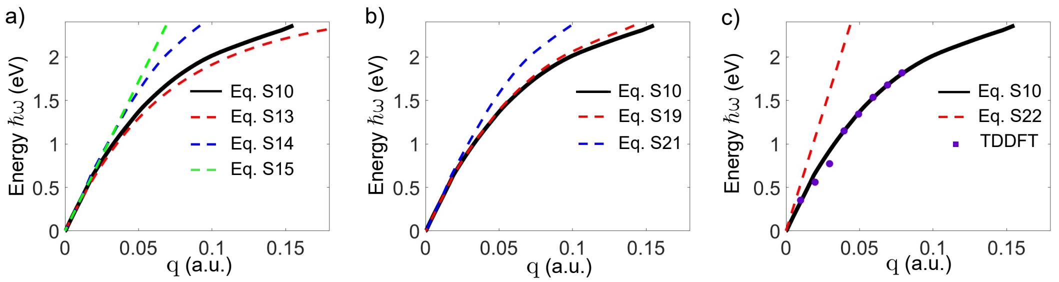

Figure S5: Non-retarded dispersion relation of the system sketched in Figure S1, as given by the equations indicated in the legend, with $\delta = 2$ Å. The results in (a) and the black line in (b,c) refer to the classical dipersion of the MIMI system in Figure S1a. The classical dispersion shown by the dashed lines in (b) are obtained for the system in Figure S1b, where the thin metal layer is substituted by a 2D layer of conductivity σ_{2D}. Last, in (c), the red-dashed dispersion corresponds to the acoustic surface plasmon of the system according to Eq. (S22) and the violet dots to the TDDFT results (the latter extracted from Figure S3d and corresponding to the dots of the same color in Figure 2e of the main text). The classical results are obtained using the gold experimental permittivity [1] (extended towards lower energies using a Drude-like frequency dependence) except for the dashed red and green line in (a), where a Drude-model description is used ($\varepsilon_\infty = 8.5$, $\lambda_p = 148$ nm). Eq. (S10) and Eq. (S19) are solved for each energy by numerical error minimization with a complex q.

S2.1 Approximated dispersion

S2.1.1 First approximated dispersion

The first approximation for the dispersion can be obtained by modelling the gold permittivity as a modified Drude permittivity

$$\varepsilon_{\mathrm{Au}} = \varepsilon_\infty - \frac{\omega_p^2}{\omega^2} = \varepsilon_\infty - \frac{\lambda^2}{\lambda_p^2}, \tag{S11}$$

where we neglect losses, $\varepsilon_\infty = 8.5$ is the background permitivity, ω is the (angular) wavelength, λ is the vacuum wavelength, ω_p is the plasmon frequency, and $\lambda_p = 2\pi c_0/\omega_p = 148$ nm is the plasma wavelength for Au (c_0 speed of light in vacuum). Inserting Eq. (S11) into Eq. (S10) and defining $f = \left[1 - \left(1 - e^{-2q\delta}\right)\left(1 - e^{-2qs}\right)\right]^{-\frac{1}{2}}$, we obtain

$$1 - \left(\frac{\varepsilon_\infty - \frac{\lambda^2}{\lambda_p^2} - \varepsilon_{\mathrm{d}}}{\varepsilon_\infty - \frac{\lambda^2}{\lambda_p^2} + \varepsilon_{\mathrm{d}}}\right)^2 f^{-2} = 0, \tag{S12}$$

which can be written as

$$\lambda_{\mathrm{MIMI}} = \lambda_p \sqrt{\varepsilon_\infty + \varepsilon_{\mathrm{d}} \frac{f+1}{f-1}}, \tag{S13}$$

corresponding to Eq. (1) in the main text. The dispersion obtained with this equation (using $\omega = 2\pi c_0/\lambda$) is given by the dashed red line in Figure S5a and is in very good agreement for energy $\gtrsim 0.075$ eV with the exact non-retarded dispersion given by Eq. (S10) (black line).

S2.1.2 Second approximated dispersion

As a second approximation, we consider again the experimental gold permittivity [1], expand the exponentials in Eq. (S10) to first order, $e^{-2q\delta} \approx 1 - 2q\delta$ and $e^{-2qs} \approx 1 - 2qs$, and obtain

$$q \approx \sqrt{\frac{-\varepsilon_{\rm Au}\varepsilon_{\rm d}}{\left(\varepsilon_{\rm Au} - \varepsilon_{\rm d}\right)^2 s\delta}}. \tag{S14}$$

This dispersion is shown by the dashed blue line in Figure S5a. The result is in good agreement with the exact solution of Eq. (S10) (black line) for low energies. The agreement significantly worsens for energies larger than $\gtrapprox$ 1 eV, with Eq. (S14) giving a smaller q for the same energy as Eq. (S10), although the difference remains moderate.

S2.1.3 Third approximated dispersion

Considering that at low energy Eq. (S11) becomes $\varepsilon_{\rm Au} \approx -\lambda^2/\lambda_p^2 \gg \varepsilon_{\rm d}$, we can further simplify Eq. (S14) and write

$$q \approx \sqrt{\frac{\varepsilon_d}{s\delta}}\frac{\lambda_p}{\lambda}, \tag{S15}$$

which indicates that, for low energies, q is inversely proportional to the wavelength (i.e. linear with energy) and scales with the inverse of the square root of the gap width, $q \propto 1/\sqrt{\delta}$. The results for $\lambda_p = 148$ nm are shown by the green-dashed blue line in Figure S5a, which verifies that the simple Eq. (S15) indeed matches well the low-energy (0.075 eV$\lessapprox \hbar\omega \lessapprox$ 0.8 eV) behavior of the exact non-retarded classical dispersion (black line).

S2.1.4 Derivation of the dispersion from the retarded formalism

In this subsection, we discuss briefly how to derive Eq. (S10) using a full retarded formalism. The dispersion is given in this case by the zeros of $1 - r_f r_s e^{2ik_{\rm d,z}\delta} = 0$, where r_s is the reflection coefficient between vacuum and the semiinfinite gold substrate (the standard Fresnel coefficient $r_s = \frac{k_{d,z}\varepsilon_{\rm Au} - k_{\rm Au,z}\varepsilon_{\rm d}}{k_{\rm d,z}\varepsilon_{\rm Au} + k_{\rm Au,z}\varepsilon_d}$) and r_f is the equivalent value between vacuum and the thin metal layer. Using the approximation $k_{\rm d,z} \approx k_{\rm Au,z} \approx iq$, which is valid for large values of q, $r_s = \frac{\varepsilon_{Au} - \varepsilon_d}{\varepsilon_{Au} + \varepsilon_d}$, $r_f = r_s \frac{1 - e^{2ik_{\rm Au,z}s}}{1 - r_s^2 e^{2ik_{\rm Au,z}s}}$ and Eq. (S10) can be obtained straightforwardly.

S2.2 Dispersion when treating the metal layer as a 2D material

An alternative dispersion can be obtained by treating the thin gold layer of thickness s as an infinitesimally-thin two-dimensional (2D) material of the same total conductivity at distance δ from the metal substrate, as sketched in Figure S1b. The 3-dimensional (3D) conductivity of gold is $\sigma = -i\omega\varepsilon_0(\varepsilon - 1)$, with ε_0 the vacuum permittivity, so that the 2D material is characterized by 2D conductivity

$$\sigma_{2D} = -i\omega\varepsilon_0(\varepsilon_{\rm Au} - 1)s. \tag{S16}$$

Following the same approach as in Sec. S2.1.4, we obtain the dispersion by solving the equation $1 - r_f r_s e^{2ik_{d,z}\delta} = 0$, where r_f is now the reflection coefficient of the 2D layer (surrounded by vacuum with permittivity $\varepsilon_{\rm d} = 1$) [14, 15],

$$r_f = \frac{k_{d,z}\frac{\sigma_{2D}}{\omega\varepsilon_0}}{k_{d,z}\frac{\sigma_{2D}}{\omega\varepsilon_0} + 2\varepsilon_\mathrm{d}}. \tag{S17}$$

Last, considering again very large q and $k_{d,z} \approx k_{m,z} \approx iq$, we obtain the dispersion

$$1 - \frac{iq\frac{\sigma_{2D}}{\omega\varepsilon_0}}{iq\frac{\sigma_{2D}}{\omega\varepsilon_0} + 2\varepsilon_\mathrm{d}} r_s e^{-2q\delta} = 0, \tag{S18}$$

or

$$1 - \frac{iq\frac{\sigma_{2D}}{\omega\varepsilon_0}}{iq\frac{\sigma_{2D}}{\omega\varepsilon_0} + 2\varepsilon_\mathrm{d}} \frac{\varepsilon_{Au} - \varepsilon_d}{\varepsilon_\mathrm{Au} + \varepsilon_\mathrm{d}} e^{-2q\delta} = 0. \tag{S19}$$

We compare in Figure S5b the classical dispersion obtained when treating the upper gold layer as a 2D material (Eq. (S19), dashed red line) and as a thin layer (Eq. (S10), solid black line) and find that they are almost identical for the $\delta = 2$ Å thickness considered. The agreement worsens as the gap thickness δ is decreased (not shown), but we have checked that it remains excellent for gaps as narrow as $\delta = 1 - 1.5$ Å (the thinnest gaps where the classical description can give a reasonable description of the system before quantum effects become too strong).

S2.2.1 Fourth approximated dispersion

Eq. (S19) can be simplified at low energies, as in this case $\mid \varepsilon_{Au} \mid \gg \varepsilon_d$, which gives

$$1 - \frac{iq\frac{\sigma_{2D}}{\omega\varepsilon_0}}{iq\frac{\sigma_{2D}}{\omega\varepsilon_0} + 2\varepsilon_d} e^{-2q\delta} = 0. \tag{S20}$$

Expanding the exponential in Eq. (S20) to first order , we obtain the approximate dispersion

$$q \approx \sqrt{\frac{i\varepsilon_d}{\delta}\frac{\omega\varepsilon_0}{\sigma_{2D}}}, \tag{S21}$$

which corresponds to the dashed blue line in Figure S5b. This dispersion agrees reasonably well with the exact non retarded results (Eq. (S10), black line), particularly at low energies, where the match is excellent. All results in Figure S5b are obtained using the experimental gold permittivity, but for a Drude description $\varepsilon_{Au} = \varepsilon_\infty - \frac{\omega_p^2}{\omega^2}$ with $\varepsilon_\infty = 1$, Eq. (S16) can be written as $\frac{\sigma_{2D}}{\omega} = i\varepsilon_0\frac{\omega_p^2}{\omega^2}s = i\varepsilon_0\frac{\lambda^2}{\lambda_p^2}s$, and Eq. (S21) becomes identical to Eq. (S15).

S2.2.2 Fifth approximated dispersion

The dispersion of the MIMI can also be derived following a different approach [16], that considers the response function of a 2D sheet in vacuum placed far outside the 3D substrate derived for a 2D electron gas [17]. The resulting dispersion of the low-energy plasmon (corresponding to an acoustic mode) is expressed at small momentum transfers (small q) by [16, 18, 19]

$$\omega = v_F q\sqrt{1 + 16\frac{\delta^2}{1 + 8\delta}}, \tag{S22}$$

where $v_F = 0.69/137c_0$ is the Fermi velocity of the metal, c_0 the speed of light in vacuum and the separation distance δ between the 2D sheet and the 3D substrate is written in atomic units

[16]. The numerical results are given by the dashed red line in Figure S5c. The agreement with the exact classical non-retarded results given by Eq. (S10) (black line) is only moderate.

Last, the violet dots in Figure S5c represent the dispersion obtained from the TDDFT calculation (same results as given by the dots of the same color in Figure 2e in the main text). It agrees very well with the results obtained with Eq. (S10) over all the frequency range considered, and significantly less well with those obtained with Eq. (S22). Thus, we find that the dispersion is very well described by the simple approach of directly calculating the classical response with the (local) experimental permittivity (Eq. (S10)).

S2.3 Dispersion of MIM structure and surface plasmon

The non-retarded dispersion of a Metal-Insulator-Metal (MIM) structure formed by a dielectric gap of thickness d and permittivity ε_{d} between two semi-infinite gold surfaces can be obtained directly from Eq. (S10) by taking the limit $s \to \infty$ and substituting δ by d. We obtain

$$1 - \left(\frac{\varepsilon_{\mathrm{Au}} - \varepsilon_{\mathrm{d}}}{\varepsilon_{\mathrm{Au}} + \varepsilon_{\mathrm{d}}} \right)^2 e^{-2qd} = 0. \tag{S23}$$

Similarly, the approximated Eq. (S13) becomes

$$\lambda_{\mathrm{MIM}} = \lambda_p \sqrt{\varepsilon_\infty + \varepsilon_{\mathrm{d}} \frac{e^{qd} + 1}{e^{qd} - 1}}. \tag{S24}$$

However the non-retarded dispersion of the MIM given by Eqs. (S23, S24) is not accurate at low energies. The retarded expression can be found in Ref. [20], which for narrow gaps can be simplified [21] to

$$q = \frac{\omega}{c_0} \sqrt{\varepsilon_d + A \left[1 + \sqrt{1 + \frac{2(\varepsilon_d - \varepsilon_{Au})}{A}} \right]},$$
$$A = 2 \left[\frac{\varepsilon_d c_0}{\omega d \varepsilon_{Au}} \right]^2. \tag{S25}$$

Last, the retarded dispersion of the surface plasmon polariton (SPP) supported at the interface between a seminfinite gold substrate and the surrounding dielectric material of permittivity ε_{d} is given by [22]

$$q = \frac{\omega}{c_0} \sqrt{\frac{\varepsilon_{\mathrm{Au}} \varepsilon_{\mathrm{d}}}{\varepsilon_{\mathrm{Au}} + \varepsilon_{\mathrm{d}}}}. \tag{S26}$$

S3 Classical electromagnetic simulations of a NPoM with an atomic-thick patch

In this section, we consider simulations of an axially-symmetric metallic nanosystem to illustrate the effect on the optical response (and thus on the Electronic Raman Scattering ERS) of the formation of a nanopatch configuration. The system is formed by two closely situated gold nanoparticles in vacuum $\varepsilon_d = 1$ with one spherical half (with diameter 80 nm) and the other half ending in a flat facet of diameter 40 nm perpendicular to the z direction (with a rounded edge between the facet and the semispherical half of 20 nm radius). The flat facets of the two

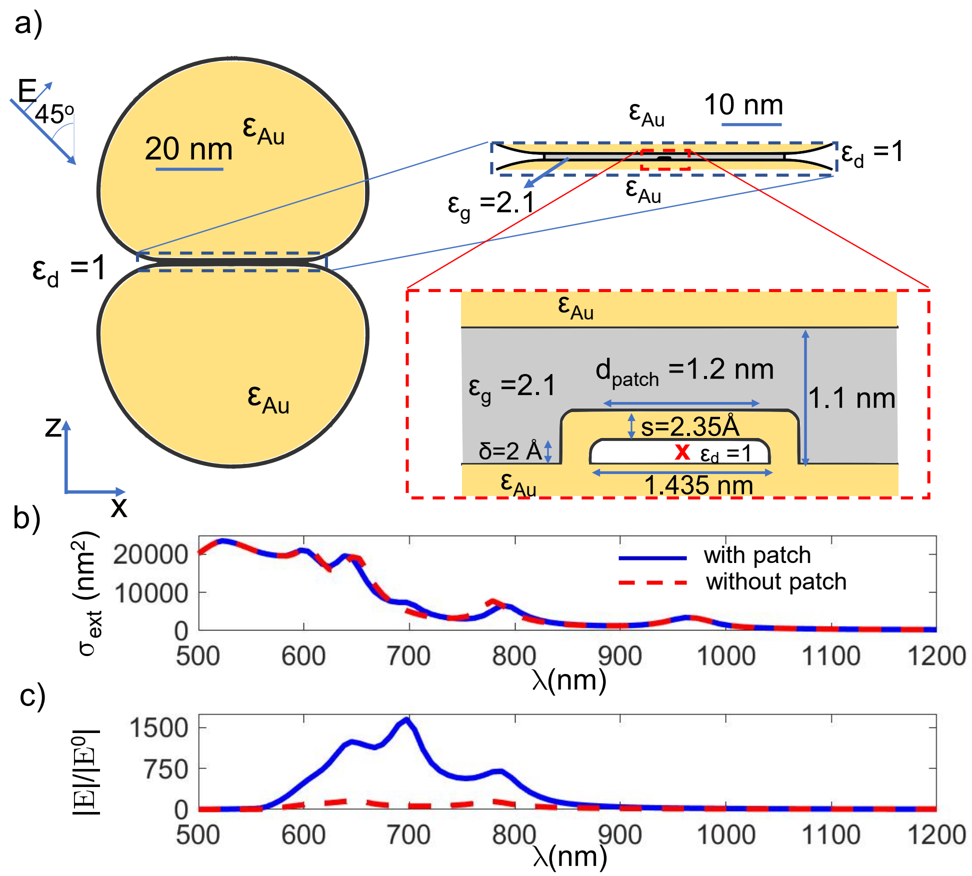

Figure S6: (a) Sketch of plasmonic nanoestructure presenting a nanopatch at the center of the 1.1 nm-thick gap of permittivity $\varepsilon_{\mathrm{g}} = 2.1$ formed between two circular flat facets of radius 20 nm of two gold particles. The patch consists of a gold monolayer (thickness $s = 2.35$ Å and diameter 1.2 nm) separated by a subnanometer slot (thickness $\delta = 2$ Å) of permittivity $\varepsilon_{\mathrm{d}} = 1$ above the facet of the bottom particle. The particles are surrounded by vacuum $\varepsilon_{\mathrm{d}} = 1$ and are spherical (with diameter 80 nm) except for the flat facets. The whole system is axially symmetric. The exact definition of these parameters and the rounding of the corners of the patch can be found in the sketch. (b) Extinction cross-section σ_{ext} spectra and (c) field enhancement $|E/E_0|$ of the system (solid blue line) and for the same system but without the patch (dashed red line). The system is illuminated by a plane wave incoming at a 45° degree angle with respect to the direction z perpendicular to the flat facets. The linear polarization is chosen so that the electric field is in the plane xz (see axis and illumination scheme in (a)). The field enhancement is evaluated in the center of the slot, in the position indicated by the red cross in the bottom-right of (a).

particles are separated by a very narrow (1.1 nm) gap of permittivity $\varepsilon_{\rm gap} = 2.1$. This system is very similar to a Nanoparticle-on-Mirror (NPoM) configuration where the bottom particle is substituted by a semi-infinite gold substrate, in which mirror charges would be induced so that it behaves as the missing particle. The details of the system can be appreciated in the sketch in Figure S6a.

The patch, of diameter $d_{patch} = 1.2$ nm, is formed by the lifting by $\delta = 0.2$ Å of a small circular section of a gold monolayer (thickness $s = 2.35$ Å) from the center of the bottom flat facet. Corners are slighly rounded to avoid divergences in the field enhancement, and the slot formed below the monolayer is also described by a permittivity $\varepsilon_d = 1$. The system is excited by a plane wave arriving at an angle of 45° to the z axis and p-polarized with respect to the flat facets (electric field vector in the xz plane, see axis of coordinates and details of the geometry, including exact definitions and rounding, in the sketch).

The extinction cross-section in Figure S6b (solid blue line) shows several peaks corresponding to the excitation of plasmonic modes. The extinction cross-section spectra is compared to that obtained from the same system but without the nanopatch (red dashed line). The difference between the two results is small, so that it would be challenging to identify the formation of the patch in an experiment from the extinction cross-section. However, it is still notable that some differences can be appreciated between the two spectra for such small patch.

The effect of the patch is dramatically larger when considering the local fields, as shown in Figure S6c. Here, the field enhancement $|E|/|E^0|$ is evaluated at the position corresponding to the center of the 2 Å thick slot formed between the facet and the lifted gold monolayer (position marked by a red cross in the bottom-right sketch of Figure S6a). We find that the maximum field enhancement when the patch is included (solid blue line in Figure S6c) is $|E|/|E^0| \approx 1650$, about 10 times larger than when it is not (red-dashed line). For reference, in the context of Surface-Enhanced Raman Spectroscopy, such enhancements would correspond to an enhancement $\gtrapprox 5 \cdot 10^{12}$ of the Raman signal of a molecule (if we ignore the spatial inhomogeneity of the field and that the slot is too small to contain most molecules of interest). Such huge enhancements are possible due to extreme localization of the fields, consistent with recent work [23] that discusses modes with effective volume < 1 nm^3 near atomic-sized tips (picocavities). Importantly, the fields are also enhanced inside the metal at and near the patch (not shown), which would lead to strong increase of the ERS signal (or other light emission from Au) and thus explain the measured flares.

These results support the hypothesis that the formation of such nanopatches is the reason behind the experimental flares, which is remarkable when considering that the patches are composed of only a few atoms. We note, however, that additional calculations (not shown) suggest that the field enhancement is extremely sensitive to the exact shape and dimensions of the patch and of the rest of the structure. Further, the classical calculations may neglect losses due e.g. to additional electron scattering in the atomic monolayer. Thus, a reliable estimation of the field enhancement and localization achievable requires further work.

S4 Experimental measurement of flares

In this section we give further details of the experiments shown in the main text. We utilise the well-known nanoparticle-on-mirror (NPoM) geometry which is highly effective at impedance-matching free space electromagnetic fields through the nanoparticle antenna into a nm-wide gap between nanoparticle and underlying mirror (see [10] in main text). While a wide variety of spacer molecules have been explored [24], here we employ one of the most stable molecules, biphenylthiol (BPT), which forms rigid robust self-assembled molecular monolayers (SAMs) of thickness 1.1 nm. Monodisperse 80 nm diameter Au nanoparticles are used (BBI), with typical quasi-circular facets of radius 10 nm. The dark field scattering spectra of many thousand NPoMs are recorded on every sample (using an automated microscope system and software), to check that the lowest-energy coupled plasmon resonance of each nanocavity is centered at the correct

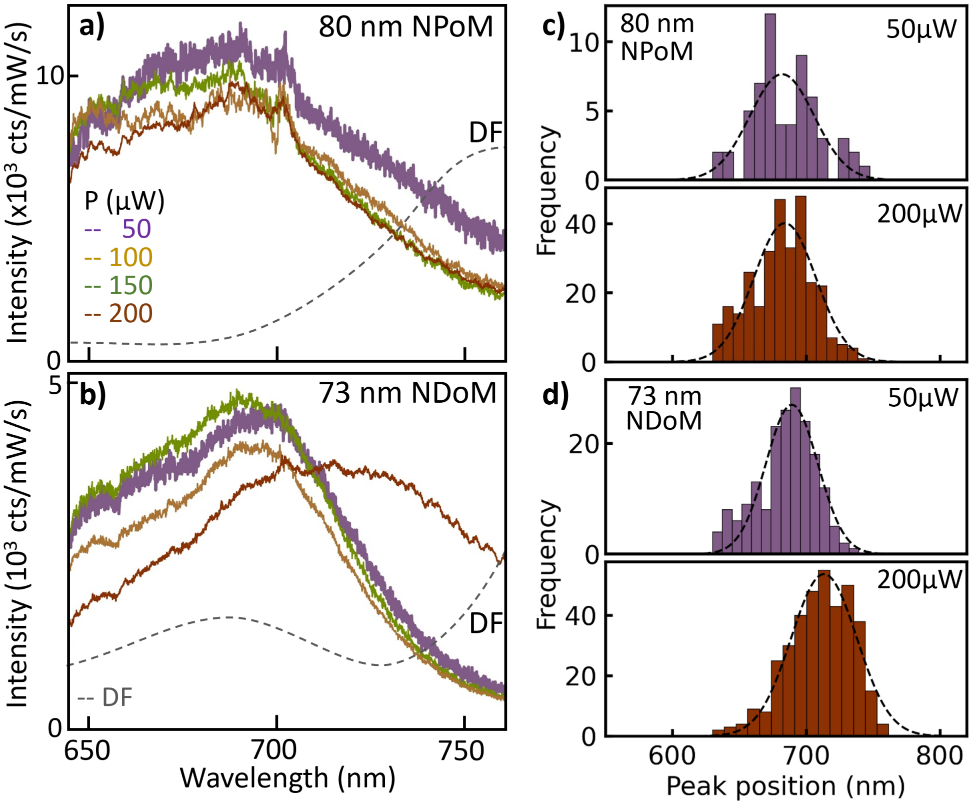

Figure S7: Average flare spectra (after removing SERS lines) vs average 633 nm laser power (colours as in legend) from (a) spherical NPoM and (b) decahedral NDoM nanoparticle-on-mirror constructs. Corresponding NPoM dark-field spectra shown (grey dashed line). (c,d) Statistics of flare peak wavelengths (Gaussian fits) for many events and low and high laser powers.

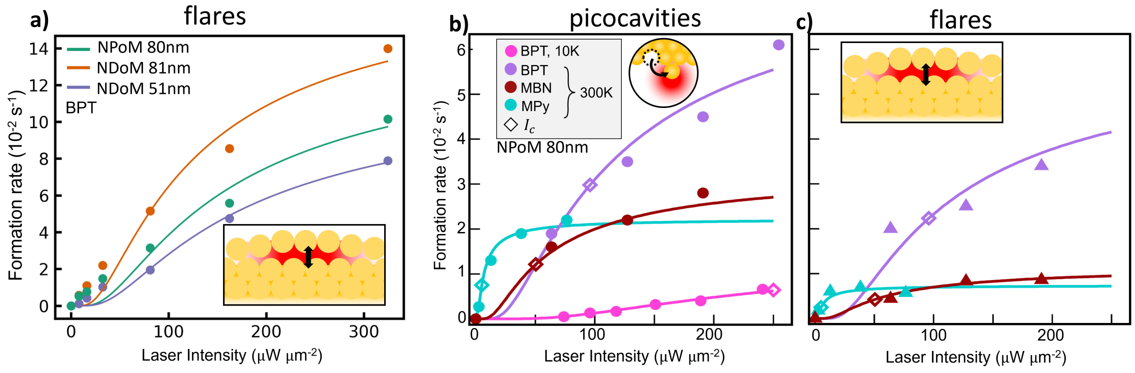

Figure S8: (a) Average flare generation rate vs laser power for 3 separate samples of NPoMs and NDoMs. The NPoM diameter is 80 nm, while the triangular facets for the NDoMs are 51 nm and 81 nm. (b) Picocavity generation rate vs power for a range of molecular SAMs using 80 nm NPoMs from Ref. [24]. Biphenyl-4-thiol (BPT; purple), 4-mercaptobenzonitrile (MBN; brown), and 4-mercaptopyridine (MPy; cyan). (c) Flare generation rate vs power for same range of molecular SAMs as in panel (b). Fits in all cases use the same formula, described in Ref. [24].

wavelength of 790 nm (Figure 3b dashed).

Measurements of the inelastic scattering are performed by focussing 50-200 μW of 633 nm pump light using a $\times$100 microscope objective onto an individual NPoM, and recording the scattered light after filtering out the laser. At low pump powers, only the SERS of the BPT molecules is observed over many minutes [24]. At higher powers, after some time delay, new lines start to appear as well as broader spectral features. We separate these into picocavity events (when additional sharp vibrational lines are seen, which fluctuate in intensity and energy) and flare events [24, 25]. The latter correspond to changes in the background of inelastic emission, which are discussed in the text. We note that using a notch filter to remove the pump light shows that some of the inelastic emission appears on the antiStokes (higher energy) side of the pump (Figure 3b).

The k-space imaging is performed by focussing the back-focal plane of the objective lens onto a CCD camera, with notch filters to remove all laser light. This spectrally integrates all emission and includes the SERS of the BPT as well (which is strongest at high angles). Images of the constant nanocavity emission in time (SERS and constant background) are recorded initially, and then subtracted from the data to leave only the resulting flare emission (as seen in Figure 3b).

The power dependence of the flares is recorded for samples both using near-spherical nanoparticles, and with those using decahedra to form nanoparticle-on-decahedra constructs (NDoM, see [47] of main text for full details and spectroscopy characterisation). While decahedra only possess triangular (111) facets, the spherical nanoparticles show a combination of square (100) and triangular (111) facets, which vary over a wide range of facet diameters. Statistics on many NPoMs and NDoMs are recorded, extracting both the average flare spectra (Figure S7a,b) as well as the histogram of the central wavelength of each flare spectrum (Figure S7c,d).

From these data it is possible to extract the statistics of flare formation rates and compare them to picocavity formation rates (see Ref. [24]). In all cases, at lower powers flares cannot form, and above a critical intensity threshold the rate rises steeply, saturating at higher powers (Figure S8a). We find that the same model of light-induced reduction of barrier for formation of both picocavities and flares can fit all data (lines, Figure S8a-c). The effect of the polarizability of the molecules on the flare production rate is to change both the final maximum formation rate, and the intensity threshold (Figure S8c).

References

[1] P. B. Johnson and R. W. Christy. Optical Constants of the Noble Metals. Phys. Rev. B **6**, 4370-4379 (1972).

[2] B. N. J. Persson and E. Zaremba. Electron-Hole Pair Production at Metal Surfaces. Phys. Rev. B **31**, 1863-1872 (1985).

[3] L. Vattuone, M. Smerieri, T. Langer, C. Tegenkamp, H. Pfnür, V. M. Silkin, E. V. Chulkov, P. M. Echenique, and M. Rocca, Correlated Motion of Electrons on the Au(111) Surface: Anomalous Acoustic Surface-Plasmon Dispersion and Single-Particle Excitations. Phys. Rev. Lett. **110**, 127405 (2013).

[4] D. M. Ceperley and B. J. Alder, Ground State of the Electron Gas by a Stochastic Method. Phys. Rev. Lett. **45**, 566–569 (1980).

[5] V. M. Silkin, E. V. Chulkov, I. Y. Sklyadneva, and V. E. Panin, Self-Consistent Calculation of the Electron Energy Spectrum of Aluminum. Sov. Phys. J. **27**, 762–767 (1984).

[6] N. Troullier and J. L. Martins, Efficient Pseudopotentials for Plane-Wave Calculations. Phys. Rev. B **43**, 1993–2006 (1991).

[7] K. Glantschig and C. Ambrosch-Draxl, Relativistic Effects on the Linear Optical Properties of Au, Pt, Pb and W. New J. Phys. **12**, 103048 (2010).

[8] E. E. Krasovskii, A. N. Yaresko, and V. N. Antonov, Theoretical Study of Ultraviolet Photoemission Spectra of Noble Metals. J. Electron Spectros. Relat. Phenomena **68**, 157–166 (1994).

[9] E. Runge and E. K. U. Gross, Density-Functional Theory for Time-Dependent Systems. Phys. Rev. Lett. **52**, 997–1000 (1984).

[10] M. Petersilka, U. J. Gossmann, and E. K. U. Gross, Excitation Energies from Time-Dependent Density-Functional Theory. Phys. Rev. Lett. **76**, 1212–1215 (1996).

[11] A. Alkauskas, S. D. Schneider, C. Hebert, S. Sagmeister, and C. Draxl, Dynamic Structure Factors of Cu, Ag, and Au: Comparative Study from First Principles. Phys. Rev. B **88**, 195124 (2013).

[12] V. M. Silkin, E. V. Chulkov, and P. M. Echenique, First-Principles Calculation of the Electron Inelastic Mean Free Path in Be Metal. Phys. Rev. B **68**, 205106 (2003).

[13] V. M. Silkin, E. V. Chulkov, and P. M. Echenique, Band Structure versus Dynamical Exchange-Correlation Effects in Surface Plasmon Energy and Damping: A First-Principles Calculation. Phys. Rev. Lett. **93**, 176801 (2004).

[14] F. H. L. Koppens, D. E. Chang, and F. J. García de Abajo. Graphene Plasmonics: A Platform for Strong Light–Matter Interactions. Nano Lett. **11**, 3370-3377 (2011).

[15] M. Ameen Poyli, M. Hrtoň, I. A. Nechaev, A. Y. Nikitin, P. M. Echenique, V. M. Silkin, J. Aizpurua, and R. Esteban. Controlling Surface Charge and Spin Density Oscillations by Dirac Plasmon Interaction in Thin Topological Insulators. Phys. Rev. B **97**, 115420 (2018).

[16] J. M. Pitarke, V. U. Nazarov, V. M. Silkin, E. V. Chulkov, E. Zaremba, and P. M. Echenique, Theory of Acoustic Surface Plasmons. Phys. Rev. B **70**, 205403 (2004).

[17] F. Stern, Polarizability of a Two-Dimensional Electron Gas. Phys. Rev. Lett. **18**, 546-548 (1967).

[18] S. Das Sarma and A Madhukar, Collective Modes of Spatially Separated, Two-Component, Two-Dimensional Plasma in Solids. Phys. Rev. B **23**, 805-815 (1981).

[19] J. M. Pitarke, V. M. Silkin, E. V. Chulkov, and P. M. Echenique, Theory of Surface Plasmons and Surface-Plasmon Polaritons. Rep. Prog. Phys. **70**, 1-87 (2007).

[20] J. A. Dionne, L. A. Sweatlock, H. A. Atwater, and A. Polman, Plasmon Slot Waveguides: Towards Chip-Scale Propagation with Subwavelength-Scale Localization. Phys. Rev. B **73**, 035407 (2006).

[21] S. I. Bozhevolnyi, and T. Søndergaard, General Properties of Slow-Plasmon Resonant Nanostructures: Nano-Antennas and Resonators. Opt. Express **15**, 10869-10877 (2007).

[22] H. Raether. Surface Plasmons on Smooth and Rough Surfaces and on Gratings. Springer Tracts in Modern Physics, Springer-Verlag Berlin: Heidelberg, 1988.

[23] T. Wu, W. Yan, and P. Lalanne. Bright Plasmons with Cubic Nanometer Mode Volumes through Mode Hybridization. ACS Photonics **8**, 307-314 (2021).

[24] Q. Lin, S. Hu, T. Földes, J. Huang, D. Wright, J. Griffiths, E. Elliott, B. de Nijs, E. Rosta, and J. J. Baumberg, Optical Suppression of Energy Barriers in Single Molecule-Metal Binding. Sci. Adv. **8**, eabp9285 (2022).

[25] C. Carnegie, M. Urbieta, R. Chikkaraddy, B. de Nijs, J. Griffiths, W. M. Deacon, M. Kamp, N. Zabala, J. Aizpurua, and J. J. Baumberg, Flickering Nanometre-Scale Disorder in a Crystal Lattice Tracked by Plasmonic Flare Light Emission, Nat. Commun. **11**, 682 (2020).
